# Embodied groove–synchrony model: movement context reshapes groove–synchrony coupling and its dominant timescale

**DOI:** 10.3389/fpsyg.2026.1803480

**Published:** 2026-05-15

**Authors:** Hiroko Tanabe, Minami Nakajima, Mai Shiratori, Kota Yamamoto, Masahiro Okano

**Affiliations:** 1 Graduate School of Humanities and Human Sciences, Hokkaido University, Sapporo, Japan; 2 Faculty of Humanities and Human Sciences, Hokkaido University, Sapporo, Japan; 3 School of Humanities, Hokusei Gakuen University, Sapporo, Japan; 4 Graduate School of Human Development and Environment, Kobe University, Kobe, Hyogo, Japan; 5 Research Organization of Science and Technology, Ritsumeikan University, Kusatsu, Shiga, Japan

**Keywords:** auditory–motor synchronization, embodied cognition, groove experience, movement context, phase-locking value, predictive processing, rhythm perception

## Abstract

**Introduction:**

Groove—the pleasurable urge to move to music—is commonly theorized to arise from embodied engagement with rhythmic structure, particularly through entrained bodily movement. However, it remains unclear how different movement contexts shape the relationship between groove experience and auditory–motor synchronization, and whether stronger synchronization uniformly enhances groove. The present study aimed to clarify how musical syncopation, bodily movement context, and timing-specific synchronization jointly organize the experience of groove.

**Methods:**

Participants listened to musical rhythms with three levels of syncopation (low, medium, high) under three movement conditions: Free (movement neither instructed nor restricted), Static (movement restricted), and Dynamic (intentional rhythmic movement required). After each stimulus, participants rated urge-to-move and pleasure. Auditory–motor synchronization was quantified using phase-locking values (PLV) between musical rhythms and head movement at two distinct metrical levels (1 Hz and 2 Hz). To characterize the functional organization of groove across movement contexts, analyses focused on structural equation modeling, complemented by supplementary linear mixed-effects models to assess trial-by-trial associations.

**Results:**

Across all movement conditions, urge-to-move robustly increased synchronization at 2 Hz, indicating that motivational drive toward movement facilitates temporal alignment with musical rhythms even when overt movement is constrained. Critically, however, synchronization was not uniformly beneficial for groove. In Free and Dynamic conditions, increased PLV exerted negative effects on groove-related ratings, suggesting that overly rigid temporal alignment may constrain the subjective groove experience. Moreover, the functional role of synchronization differed across movement contexts: groove-related effects were primarily associated with synchronization at a slower metrical level (1 Hz) in the Free condition, whereas beat-level (2 Hz) synchronization played a dominant role in the Dynamic condition, indicating context-dependent temporal scaling of auditory–motor coupling.

**Discussion:**

These findings indicate that groove is not simply amplified by stronger synchronization. Instead, groove emerges from a context-dependent balance between entrained bodily engagement with music and the degree to which movement becomes temporally stabilized to rhythmic structure. We propose an Embodied Groove–Synchrony Model, which distinguishes entrained bodily engagement from phase-based synchronization and shows that excessive temporal stabilization can, depending on movement context, attenuate rather than enhance the groove experience.

## 1 Introduction

Music plays a fundamental role in human societies, serving not only as a source of aesthetic enjoyment but also as a powerful medium for social bonding, communication, and emotional regulation. Across cultures and historical periods, music has accompanied collective rituals, celebrations, and everyday activities, enriching human life by structuring time and coordinating shared experience. Although music is often treated as a primarily auditory phenomenon, everyday musical engagement frequently extends beyond passive listening. In contexts such as concerts, clubs, or even solitary listening, people often find themselves spontaneously and involuntarily moving their bodies—nodding their heads, tapping their feet, or dancing—synchronized with the music (Gonzalez-Sanchez et al., 2018; Zelechowska et al., 2020). Such responses highlight that musical experience is not confined to perception alone but is deeply embodied, involving the dynamic coupling of auditory perception, affect, and bodily movement (Leman and Maes, 2015; Russo, 2019). Among the various forms of embodied musical engagement, the experience commonly referred to as groove is particularly striking, as it captures the compelling tendency of certain music to invite bodily movement and sustained engagement, making it a central phenomenon for understanding how music is perceived, felt, and enacted in everyday life (e.g., Janata et al., 2012; Stupacher et al., 2022a).

The groove experience has been described using a wide range of terms, reflecting its multifaceted nature. Previous studies have characterized groove as movement-inducing (Madison, 2006), pleasurable (Janata et al., 2012), immersive (Duman et al., 2024), related to dynamic patterns of tension and relaxation (Hosken, 2020), and capable of fostering feelings of solidarity and social cohesion in musical interaction (Monson, 2009). Given this diversity of perspectives, it has been argued that there is no single, universally accepted definition of groove (Hosken, 2020). These perspectives are also consistent with prior reviews of groove and related sensorimotor synchronization and entrainment processes (e.g., Janata et al., 2012; Repp and Su, 2013). Nevertheless, syntheses of psychological and neuroscientific research converge on a core set of features. As summarized by Etani et al. (2024), groove is consistently associated with rhythm- and tempo-dependent induction of bodily movement, often characterized by bouncing-like motion, and accompanied by pleasurable affect and a sense of unity. Accordingly, empirical studies have increasingly adopted an operational definition of groove as the pleasurable sensation of wanting to move the body to music, emphasizing both movement induction and pleasure as its defining components (Janata et al., 2012; Matthews et al., 2019; Stupacher et al., 2022a; Vuust et al., 2022; Witek et al., 2014, 2020). Importantly, these two components are unlikely to be independent: both movement-related motivation and musical pleasure are associated with dopaminergic activity, implicating reward-related brain regions such as the nucleus accumbens, caudate nucleus, and medial orbitofrontal cortex (Salimpoor et al., 2011, 2015; Matthews et al., 2020). Beyond reward processing, recent theoretical and empirical work highlights a central role of prediction in the groove experience (e.g., Vuust et al., 2022; Patel and Iversen, 2014). Motor-related brain regions, including premotor cortex, supplementary motor area, and basal ganglia, are known to contribute to temporal prediction of musical beats, as formalized in frameworks such as the Action Simulation for Auditory Prediction hypothesis and predictive coding accounts (Patel and Iversen, 2014; Grahn and Rowe, 2013; Vuust et al., 2022). From this perspective, the urge to move elicited by groove is not merely an epiphenomenon but a functional response that supports the minimization of temporal prediction error, positioning groove as a phenomenon at the intersection of movement, pleasure, and predictive engagement with musical structure.

Empirical studies have shown that the groove experience is shaped by a range of musical features, particularly those related to rhythm, such as tempo, beat salience, event density, microtiming, and syncopation, as well as acoustic properties including harmonic complexity and the presence of low-frequency bass sounds (e.g., Witek et al., 2014; Janata et al., 2012). Groove evaluations further vary across musical styles, reflecting systematic differences in how these features are combined and expressed (Etani et al., 2024). Among these factors, syncopation has attracted particular attention and remains one of the most intensively studied musical determinants of groove. Syncopation is commonly understood as the placement of rhythmic events that shift or violate expected metrical accents—either formally defined in terms of metrical weight assignment (Longuet-Higgins and Lee, 1984) or more broadly as deviations from listeners’ metric expectations (Witek et al., 2014). A seminal study by Witek et al. (2014) demonstrated that groove ratings exhibit an inverted U-shaped relationship with a computational measure of syncopation, indicating that rhythms with moderate levels of syncopation elicit the strongest urge to move and pleasure. This pattern has since been replicated across a variety of stimulus types, including piano-based rhythmic patterns and drum sequences, providing robust support for the idea that syncopation is most effective when it moderately violates temporal expectations (Matthews et al., 2019, 2020, 2022; Sioros et al., 2014; Spiech et al., 2022; Stupacher et al., 2022b). Psychological accounts have interpreted this effect in terms of complementary roles of temporal regularity and temporal interest. Musical features that facilitate beat perception—such as stable tempo, salient beats, bass sounds, and dense rhythmic events—support the formation of internal representations of temporal regularity, whereas features like syncopation selectively disrupt these expectations and thereby increase interest in the temporal organization of the music (Senn et al., 2023). A closely related explanation is provided by predictive coding frameworks, which conceptualize music perception and action as coupled processes aimed at minimizing prediction error through either updating internal models or engaging bodily movement (Vuust and Witek, 2014; Vuust et al., 2018, 2022). From this perspective, rhythms with moderate syncopation maximize precision-weighted prediction error—the product of the magnitude of expectation violation and the reliability of the metrical model—thereby motivating listeners to reduce this error by moving their bodies and adjusting sensory precision. In contrast, rhythms with low syncopation generate little prediction error, whereas highly syncopated rhythms undermine metrical precision, resulting in weaker engagement in both cases. Together, these accounts converge on the idea that syncopation plays a central role in groove precisely because it occupies a critical middle ground between predictability and violation, optimally engaging perceptual, motor, and affective processes (e.g., Witek et al., 2014; Vuust et al., 2022).

Building on accumulating empirical findings, several models have been proposed to account for how musical structure gives rise to the groove experience, integrating psychological, neurocognitive, and computational perspectives. Among these, Senn et al.’s (2023) psychological model offers one of the most comprehensive accounts, proposing that the urge to move does not arise directly from musical features but is mediated by internal representations of temporal regularity, time-related interest, pleasure, and energetic arousal. In this framework, synchronized body movement plays a crucial role through multiple feedback loops: sensory feedback enhances the clarity of temporal regularities, hedonic feedback increases pleasure through movement itself, and energetic feedback elevates arousal, collectively reinforcing the groove experience. While this model successfully explains a wide range of empirical findings, Etani et al. (2024) highlight several unresolved issues. Notably, some hypothesized pathways—such as the role of time-related interest—have received limited empirical support, and in certain cases musical features appear to influence groove without following the proposed mediation structure. Moreover, although urge to move and pleasure are consistently found to be strongly correlated (Senn et al., 2020; Düvel et al., 2021; Kawase et al., 2025), their causal relationship remains unclear, with evidence suggesting that they are tightly coupled yet partially dissociable components of groove.

Complementing psychological accounts, computational models grounded in predictive coding provide a mechanistic explanation for why specific musical features, particularly syncopation, are effective in eliciting groove. These models propose that perception and action jointly minimize prediction error by either updating internal temporal models or engaging bodily movement to generate predicted sensory input (Vuust et al., 2018, 2022). Within this framework, groove is most strongly evoked when rhythms produce large precision-weighted prediction errors, as is the case with moderate levels of syncopation. Importantly, however, Etani et al. (2024) note that predictive coding models have so far primarily addressed why groove emerges under certain musical conditions, rather than how movement–music coupling dynamics unfold across different bodily contexts or time scales. Together, these perspectives converge on the idea that groove emerges from dynamic interactions between musical structure, bodily movement, and affective experience, yet they also reveal critical gaps. In particular, existing models do not specify how the strength of synchronization between movement and music, or its expression at different temporal scales, contributes to the regulation of urge to move and pleasure. Nor do they resolve whether movement-related synchronization merely facilitates groove or can also constrain or reorganize it. Against this background, the present study aims to extend current models by explicitly incorporating quantitative indices of movement–music synchronization (phase-locking values) at multiple temporal scales into a structural framework. By doing so, we seek to clarify how synchronization strength relates to urge to move and pleasure, and to shed new light on the unresolved relationship between these two core components of the groove experience.

A growing body of evidence demonstrates that the groove experience is closely intertwined with bodily movement, revealing a fundamentally bidirectional relationship between perception and action. Listening to high-groove music reliably increases spontaneous movement, particularly head movements, compared to low-groove music (Janata et al., 2012; Dotov et al., 2021), and the energetic magnitude of such movements has been shown to correlate positively with subjective groove ratings (Hurley et al., 2014). Beyond spontaneous movement, high-groove music also enhances the precision of intentional rhythmic actions: tapping accuracy improves when listening to high-groove stimuli (Janata et al., 2012), correlates positively with groove ratings (Matthews et al., 2022), and stronger synchronization between head movements and musical beats is likewise associated with higher groove evaluations (Hurley et al., 2014). This movement–music coupling extends to whole-body actions such as walking, where high-groove music increases synchronization with auditory stimuli and modulates gait patterns and balance control (Leow et al., 2014, 2021; Ready et al., 2019, 2022). Together, these findings underscore that groove is not merely a perceptual judgment but emerges from reciprocal interactions between auditory input and motor engagement. Crucially, the bidirectionality of this relationship is evident even when overt movement is minimized. Ross et al. (2016) demonstrated that when participants were instructed to stand still while listening to music, the temporal structure of their postural sway aligned with different musical time scales depending on the experienced groove level: high-groove music entrained shorter, more local temporal fluctuations in the center-of-pressure signal, whereas low-groove music was associated with entrainment to longer, more global time scales. This suggests that groove experience may actively modulate the temporal organization of motor coordination, rather than simply reflecting the amount of movement. Such findings resonate with embodied cognition accounts, which emphasize that bodily states and sensorimotor processes are integral to perception, meaning-making, and conceptual understanding (Varela et al., 2017; Fincher-Kiefer, 2019). In the context of music, embodied music cognition posits that the human motor system and bodily actions play a constitutive role in music perception, and that this influence is inherently bidirectional—encompassing both how music shapes bodily engagement and how action feeds back into auditory experience (Leman and Maes, 2015).

Despite this accumulating evidence, it remains unclear how different movement contexts—ranging from spontaneous, unconscious motion to deliberate, task-driven actions such as tapping or walking—systematically shape the relationship between groove experience, auditory–movement synchronization, and the qualitative properties of movement itself. Specifically, existing studies have yet to clarify whether changes in synchronization strength and temporal scale reflect a common underlying groove mechanism or context-dependent reconfigurations of perception–action coupling. Addressing this gap is essential for understanding how groove emerges from embodied interaction rather than from musical structure or subjective experience alone.

In the present study, “groove” was operationalized through subjective ratings of urge to move and pleasure, consistent with prior literature. The present study aims to clarify how bodily movement and movement–music synchronization contribute to the groove experience by explicitly focusing on the strength and temporal scale of synchrony. Although existing psychological and computational models of groove, such as that of Senn et al. (2023), emphasize prediction, pleasure, and urge to move, they remain underspecified regarding how sensorimotor coupling unfolds across different temporal scales and movement contexts. To address this gap, we propose an Embodied Groove–Synchrony Model, which conceptualizes groove as an emergent phenomenon arising from bidirectional interactions between auditory prediction, bodily movement, and the strength of movement–music synchronization at multiple temporal scales. Within this framework, synchronization is operationalized using phase-locking values (PLV) computed at distinct time scales, allowing us to examine how different modes of temporal alignment relate to subjective groove experience. Here, synchronization strength refers to the degree of phase consistency between movement and a reference temporal structure (i.e., canonical periodic signals at 1 Hz or 2 Hz), and temporal scales correspond to distinct frequency components (1 Hz and 2 Hz) used to quantify synchronization. Specifically, we test whether stronger synchronization is associated with higher ratings of urge to move and pleasure, and whether these associations differ across temporal scales. Furthermore, by treating urge to move and pleasure as separable yet interacting components of groove, we explore their directional relationship within the proposed model. In addition to between-condition analyses, supplementary linear mixed-effects analyses were conducted to assess whether these relationships are also present at the within-subject, trial-by-trial level. Through this approach, the present study seeks to advance existing groove theories by providing a more precise, embodied account of how groove emerges through dynamic perception–action coupling.

## 2 Methods

The study was conducted in accordance with the Declaration of Helsinki and was approved by the institutional ethics committee of Center for Experimental Research in Social Sciences, Hokkaido University (approval number: R6-24). All participants were informed about the purpose and procedures of the experiment and provided written informed consent prior to participation. Participants were informed that their participation was voluntary and that they could withdraw from the study at any time without penalty. All data were anonymized prior to analysis to ensure participant confidentiality.

### 2.1 Experimental protocols and measurements

Thirty healthy Japanese university students participated in the experiment (18 males, 12 females; mean age = 20.9 ± 1.4 years). Musical experience was assessed using the Goldsmiths Musical Sophistication Index (Gold-MSI; Müllensiefen et al., 2014; Sadakata et al., 2023), from which five factor scores (F1: active engagement; F2: perceptual abilities; F3: musical training; F4: singing abilities; F5: emotions) were computed. The Japanese version of the Gold-MSI (Sadakata et al., 2023), which has been validated for use in Japanese populations, was used. Participants exhibited moderate levels of musical experience; mean (±SD) scores were 41.77 ± 5.64 for F1 (theoretical range: 9–63), 45.20 ± 6.66 for F2 (theoretical range: 9–63), 29.17 ± 5.35 for F3 (theoretical range: 7–49), 28.47 ± 5.74 for F4 (theoretical range: 7–49), and 34.27 ± 4.33 for F5 (theoretical range: 6–42). None of the participants had any formal dance experience. All participants reported no history of visual or auditory impairments, nor any orthopedic or neurological disorders related to motor function. Regarding musical preferences, J-pop (*n* = 10) and rock (*n* = 13) were the most frequently reported favorite genres.

Auditory stimuli were based on the set of 15 syncopated rhythmic patterns used in the study by Witek et al. (2017). All stimuli were drum-based rhythmic patterns without melody or harmony, minimizing tonal influences. Using MuseScore Studio 4, MIDI-based drum sequences were created to reproduce these patterns. Each stimulus consisted of a two-bar syncopated rhythmic pattern composed of hi-hat, snare drum, and bass drum sounds, presented at a fixed tempo of 120 beats per minute (bpm), which has been identified as an optimal tempo range for eliciting strong groove experiences (Etani et al., 2018). The two-bar pattern was repeated continuously for 16 s, resulting in an eight-bar stimulus duration. The 15 rhythmic patterns were categorized into three levels of syncopation strength: low (Low), medium (Middle), and high (High), with five distinct patterns in each level. Syncopation levels (Low, Medium, and High) were defined based on the degree to which rhythmic events deviated from expected metrical positions (Witek et al., 2017). To reduce participant fatigue and limit the total number of trials, not all 15 patterns were presented to each participant. Instead, for each participant, three patterns were randomly selected from each syncopation level, resulting in a total of nine auditory stimuli per section (each participant completed three sections in total; see “Experimental design and task”). All stimuli shared the same tempo, meter, duration, and instrumental timbre, differing only in their degree of syncopation. Stimuli were presented via loudspeakers at a comfortable listening level, and sound pressure level was kept constant across stimuli and participants. For subsequent synchronization analyses, the audio waveform of each stimulus was recorded and stored at a sampling frequency of 100 Hz. The audio signal was stored for the purpose of verifying stimulus timing and was not used in the computation of synchronization measures.

Each participant completed a total of 27 trials while seated, corresponding to a combination of three levels of syncopation strength (Low, Middle, and High), three randomly selected rhythmic patterns per syncopation level, and three body movement conditions. Participants evaluated the perceived groove of each auditory stimulus under three different body movement conditions: Free, Static, and Dynamic. In the Free condition, participants were not given any instructions regarding movement. That is, no mention of movement was made, in contrast to “free movement” conditions in previous studies where participants are explicitly instructed to move freely. In the Static condition, participants were instructed to fixate on a visual marker placed at eye level and to remain as still as possible during stimulus presentation. In the Dynamic condition, participants were instructed to move their neck joints while listening to the auditory stimulus in synchrony with the music; no specific movement amplitude, direction, or beat level was prescribed, in order to allow participants to spontaneously and consciously express their preferred movement patterns in response to the music, reflecting naturally emerging movement impulses elicited by the auditory stimulus. To prevent participants from becoming overly aware of their own movements, all participants completed the Free condition first. The remaining two conditions (Static and Dynamic) were completed in the second and third sections, with the order counterbalanced across participants. Short breaks of several minutes were provided between sections to minimize fatigue. Throughout the experiment, participants were seated on a chair, and a fixation point was placed at eye level at an approximate horizontal distance of 4 m. After each stimulus presentation, participants rated their perceived groove using the Japanese version of the Extended Groove Questionnaire (EGQ-JA; Kawase et al., 2025), consisting of six items. Participants were instructed to complete the questionnaire within 30 s after each trial.

To quantify neck joint kinematics during auditory stimulus presentation, three-dimensional motion capture was performed using an optical motion capture system (OptiTrack V100: R2; NaturalPoint, Corvallis, OR, United States) composed of six infrared cameras with a sampling frequency of 100 Hz. Spherical reflective markers (12.7 mm in diameter) were affixed to 11 anatomical landmarks: the top of the head, right and left ears, upper margin of the sternum, right and left acromion, lowest edge of the right and left ribs, the C7 vertebra, and the T8 and T12 vertebrae. Three-dimensional coordinates of all markers were estimated in offline analyses. Synchronization between auditory stimuli and motion capture data was achieved using a PowerLab data acquisition system (ADInstruments, Dunedin, New Zealand). The audio signal from the stimulus presentation computer was split into two channels: one channel was sent to the loudspeakers for stimulus presentation, and the other was simultaneously recorded by the PowerLab system. At the onset of motion capture recording, a digital trigger signal (square wave) generated by the motion capture system was also recorded by PowerLab. Both audio and trigger signals were acquired synchronously using LabChart software with sampling frequency of 100 Hz (LabChart v8; ADInstruments), enabling precise temporal alignment between the auditory stimuli and motion capture data.

### 2.2 Data analysis

#### 2.2.1 Pre-processing

Groove experience was quantified using two subcomponents—“Urge-to-Move” and “Pleasure.” For each trial, Urge-to-Move and Pleasure scores were calculated as the mean of three questionnaire items each, resulting in possible scores ranging from 0 to 6.

For motion data, three-dimensional position time series of all markers were first trimmed to correspond to the 16-s duration of each auditory stimulus. The trimmed marker coordinate time series were then low-pass filtered using a fourth-order Butterworth filter with a cutoff frequency of 5 Hz, which was determined by residual analysis. Filtering was performed using the *filtfilt* function in the MATLAB Signal Processing Toolbox to avoid phase distortion. Subsequently, based on the calculation method developed by Reed et al. (1999), joint center positions of the neck, shoulder, and thoracolumbar joints were computed for each trial from the filtered marker coordinates. Three-dimensional neck joint angle time series were subsequently calculated from the filtered joint center coordinates in combination with the head and ear marker positions. Finally, to integrate motion across the three spatial dimensions, the neck joint angle data were transformed into a one-dimensional time series by computing the Euclidean norm of the three-dimensional angular displacement.

#### 2.2.2 Synchrony measures

To quantify temporal synchrony in body motion relative to canonical temporal structure, phase-locking values (PLVs) were computed at two distinct timescales. PLVs were computed between the neck movement time series and a reference sinusoidal signal at the target frequency (1 Hz or 2 Hz). Specifically, a sinusoidal signal was generated at each target frequency, and its instantaneous phase was compared with that of the movement signal to quantify the degree of phase synchronization. Importantly, the audio signal was not directly used in the PLV computation. Instead, this approach captures alignment of movement to canonical temporal structure (e.g., beat- or metrical-level periodicity), rather than direct phase coupling to the acoustic signal. In the present study, 2 Hz corresponds to the beat level at the given tempo (120 bpm), whereas 1 Hz reflects a slower metrical level. PLV was defined as the magnitude of the circular mean of the phase difference between the movement signal and the reference sinusoidal signal across time:


$$PLV=\left|\frac{1}{N}\overset{N}{\underset{t=1}{\sum }}e^{i\left(\phi_{motion}\left(t\right)-\phi_{audio}\left(t\right)\right)}\right|$$

Where $\phi_{\mathrm{motion}}\left(t\right)$ and $\phi_{\mathrm{audio}}\left(t\right)$ denote the instantaneous phases of the motion and auditory signals at time point t , respectively, and extracted at either the slower metrical frequency (1 Hz; PLV1) or beat-level frequency (2 Hz; PLV2), and N denotes the number of time points within each trial. The instantaneous phases of the motion and auditory signals were extracted using the Hilbert transform after band-pass filtering around the target frequency (bandwidth of ± 0.3 Hz). Higher PLV values indicate stronger phase consistency, ranging from 0 (no phase locking) to 1 (perfect phase locking).

### 2.3 Statistical analysis

#### 2.3.1 Two-way ANOVA and *post hoc* comparisons

To examine how rhythmic structure and movement condition influenced subjective groove ratings (Urge-to-Move and Pleasure) and motion-related synchrony measures (PLV1 and PLV2), two-way analyses of variance (ANOVAs) were conducted with Syncopation (Low, Middle, and High) and Movement Condition (Free, Static, and Dynamic) as within-subject factors. Separate ANOVAs were performed for groove ratings and for each motion-related metric. When the assumption of normality was violated, an aligned rank transform (ART) ANOVA was conducted in R. Otherwise, ANOVA and *post hoc* multiple-comparison tests were performed in Matlab R2024b. When significant main effects or interactions were observed, post-hoc multiple comparisons were conducted using appropriate correction procedures to control for family-wise error rates. Effect sizes are reported for all main analyses, using partial eta squared (*η*^2^) for ANOVA and standardized coefficients for regression-based models.

#### 2.3.2 Correlation analysis

To explore condition-specific associations between groove experience and synchrony measures, correlation analyses were conducted separately for each combination of Syncopation and Movement Condition (3 × 3 conditions). Pearson’s correlation coefficients were computed between groove ratings (Urge-to-Move and Pleasure) and each synchrony measures (PLV1 and PLV2). This analysis aimed to capture context-dependent relationships that may be obscured when collapsing across experimental conditions.

#### 2.3.3 Structural equation modeling

Structural equation modeling (SEM) was conducted using R (lavaan package) to examine the hypothesized relationships among musical properties (syncopation magnitude), groove experience (Urge-to-Move and Pleasure), and synchronization measures (PLV1 and PLV2). The resulting model is hereafter referred to as the *Embodied Groove–Synchrony Model*. The baseline structural model was grounded in the theoretical framework proposed by Senn et al. (2019), which posits that musical properties directly influence urge-to-move and pleasure and that urge-to-move promotes motor planning, generating entrained body movement. In addition, the Senn model assumes that such movement-related processes concurrently enhance pleasure and the representation of temporal regularity, which in turn increases urge-to-move. In accordance with the original Senn model, the present SEM focused on observable and self-reportable components of the groove experience. Constructs such as *time-related interest* and *motor planning*, which are assumed to operate largely at an implicit level and were not directly measurable in the present dataset, were not explicitly included as latent variables. Instead, movement-related processes were operationalized via objective synchronization indices (PLV1 and PLV2). Moreover, the effect of body movement on urge-to-move via the representation of temporal regularity proposed in the Senn model was operationalized by directly modeling paths from objective synchronization indices (PLV1 and PLV2)—reflecting phase alignment with the auditory stimulus—to urge-to-move, thereby providing an empirical test of this theoretical mechanism. Furthermore, qualitative differences in motor planning and bodily engagement were indirectly captured through the experimental Movement conditions (Free, Static, and Dynamic), which were analyzed separately. In contrast to the Movement conditions, which were examined separately due to their role in shaping bodily engagement, syncopation level (Low, Middle, and High) was treated as a musical property reflecting qualitative differences in rhythmic structure and was therefore incorporated simultaneously within a single model using dummy variables, with the Low syncopation condition serving as the reference category.

The SEM analyses were first conducted separately for each Movement condition (Free, Static, and Dynamic) to examine whether the overall pattern of structural relationships was preserved across conditions. Subsequently, multi-group SEM was employed to formally test between-condition differences in path coefficients. In the multi-group analysis, partial invariance models were tested by selectively releasing equality constraints on individual structural paths that showed evidence of group differences. This stepwise constraint–release procedure allowed identification of which specific paths exhibited condition-dependent differences. Model fit was assessed using multiple standard fit indices, including the comparative fit index (CFI), Tucker–Lewis index (TLI), root mean square error of approximation (RMSEA), and standardized root mean square residual (SRMR). Acceptable model fit was defined as CFI and TLI values ≥ 0.90, RMSEA ≤ 0.08, and SRMR ≤ 0.08, while values of CFI and TLI ≥ 0.95 and RMSEA ≤ 0.06 were considered indicative of good fit. Standardized path coefficients (std.all), which can be interpreted as effect size estimates, are reported in the SEM figures.

To complement the SEM results, linear mixed-effects models (LME) were conducted as supplementary analyses to clarify whether SEM-supported relationships reflect trial-by-trial fluctuations within individuals or primarily capture stable differences across individuals and movement contexts. Specifically, a significant within-subject effect in the LME indicates that trial-by-trial deviations from a participant’s own mean in a predictor (e.g., Urge-to-Move) are systematically associated with corresponding deviations in the outcome variable (e.g., PLV), suggesting that the SEM path reflects individual-level, dynamically varying processes. In contrast, the absence of a within-subject effect indicates that the corresponding SEM path reflects between-subject or condition-level tendencies, such that individuals who show higher average values of a given variable (e.g., stronger synchronization) also tend to show systematically different groove experiences, even if trial-by-trial fluctuations within the same individual are not predictive. LME analyses were implemented using the lme4 and lmerTest packages in R. For each SEM-supported relationship, the dependent variable was modeled as a function of the corresponding predictor(s) and their interaction with Movement condition, with random intercepts specified for participants. These analyses were not intended to replace the SEM, but to disambiguate whether structural associations reflect individual-level, trial-by-trial dynamics or higher-level regulatory structures operating at the level of between-subject differences and/or movement conditions.

## 3 Results

### 3.1 Effects of syncopation and movement conditions on groove and motion measures

**Figure 1:**
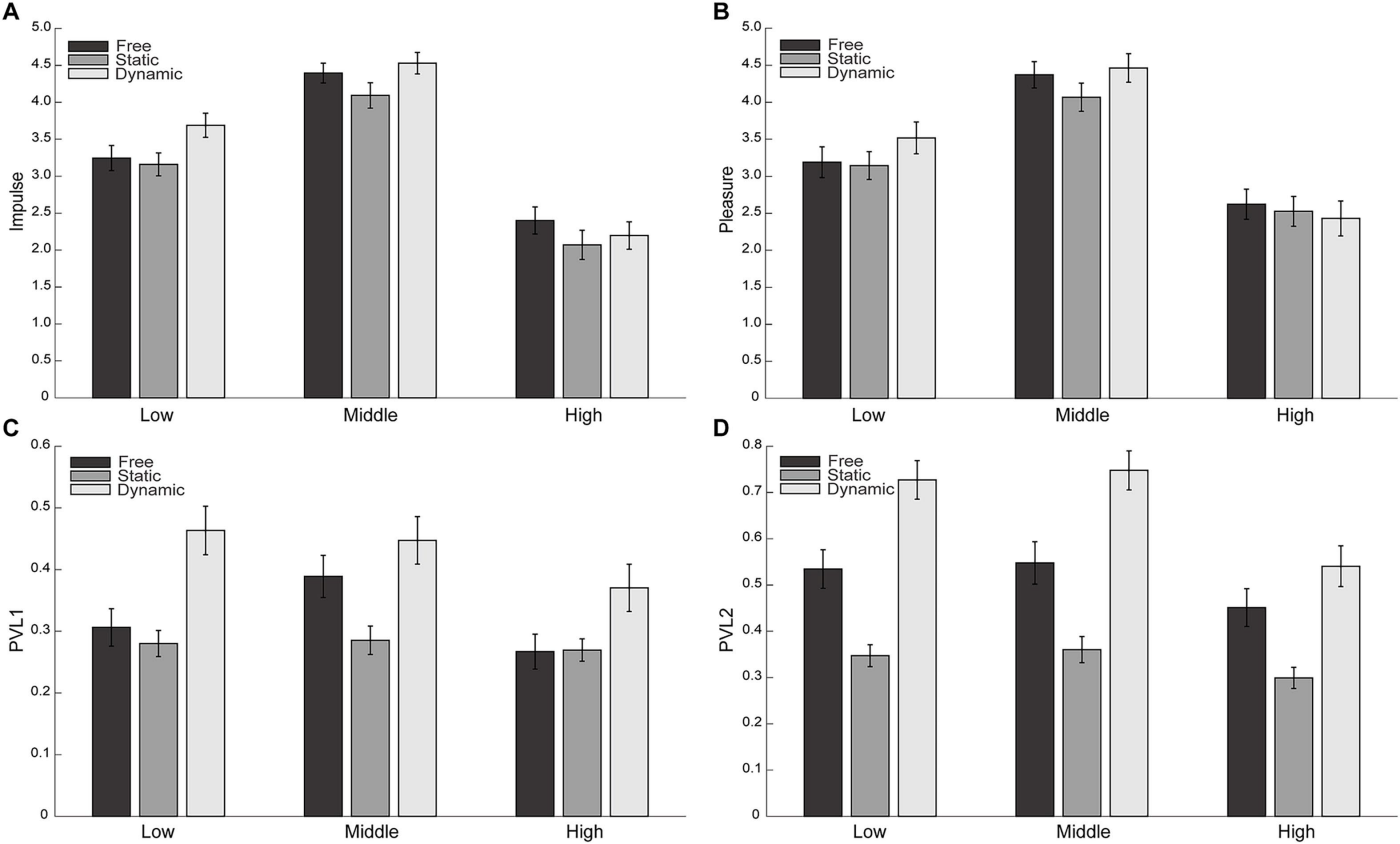
Effects of syncopation and movement condition on behavioral and synchronization measures. **(A)** Urge-to-move, **(B)** pleasure, **(C)** PLV1, and **(D)** PLV2. Bars represent mean values (± SEM) across syncopation levels (low, medium, high) for each movement condition. Movement conditions are indicated by grayscale shading: free (dark gray), static (medium gray), and dynamic (light gray).

Two-way repeated-measures ANOVAs were conducted separately for Urge-to-Move, Pleasure, PLV1, and PLV2, with Syncopation (Low, Middle, and High) and Movement condition (Free, Static, and Dynamic) as within-subject factors. An overview of the statistical results is provided in Table 1. The overall pattern of these effects is visualized in Figure 1.

**Table 1:** Full statistical results of two-way ANOVAs examining the effects of Syncopation (Low, Middle, High) and Movement condition (Free, Static, Dynamic), for groove ratings (Urge-to-Move and Pleasure) and synchrony measures (PLV1 and PLV2).

Measure	Effect	df	*F*	*p*-value	Effect size	*Post hoc* comparisons
Urge-to-Move	Syncopation	2, 261	118.7	< 0.001	ηp^2^ = 0.47	High < Low < Middle
	Movement	2, 261	3.6	0.028	ηp^2^ = 0.014	Static < Dynamic
	Interaction	4, 261	0.99	0.42	ηp^2^ = 0.0077	n.s.
Pleasure	Syncopation	2, 261	58.1	< 0.01	ηp^2^ = 0.30	High < Low < Middle
	Movement	2, 261	0.96	0.39	ηp^2^ = 0.0050	n.s.
	Interaction	4, 261	0.67	0.61	ηp^2^ = 0.0070	n.s.
PLV1	Syncopation	2, 208	8.3	< 0.0001	η^2^G = 0.84	High < Low = Middle
	Movement	2, 208	21.6	< 0.0001	η^2^G = 0.99	Static = Free < Dynamic
	Interaction	4, 208	2.6	< 0.05	η^2^G = 0.75	Descriptive trends only
PLV2	Syncopation	2, 208	14.8	< 0.0001	η^2^G = 0.96	High < Low = Middle
	Movement	2, 208	81.7	< 0.0001	η^2^G = 0.99	Static < Free < Dynamic
	Interaction	4, 208	2.3	0.058	η^2^G = 0.88	n.s.

Degrees of freedom (df), *F*-values, *p*-values, effect sizes, and *post hoc* comparisons are reported. Effect sizes are reported as partial eta squared (ηp^2^) for standard ANOVAs and as generalized eta squared (η^2^G) for aligned rank transform (ART) ANOVAs. Significant *post hoc* comparisons were conducted with appropriate corrections for multiple comparisons.

#### 3.1.1 Groove ratings

Urge-to-Move ratings showed significant main effects of Syncopation and Movement condition, with no significant interaction. *Post hoc* comparisons indicated that Urge-to-Move was highest for the Middle syncopation condition, followed by Low, and lowest for High syncopation (Middle > Low > High). Regarding Movement condition, Urge-to-Move was significantly lower in the Static condition compared with the Dynamic condition, whereas the Free condition did not differ reliably from the other conditions. For Pleasure ratings, a significant main effect of Syncopation was observed, whereas neither the main effect of Movement condition nor the interaction reached significance. Post hoc tests revealed the same ordinal pattern as Urge-to-Move, with Pleasure ratings highest for Middle syncopation, intermediate for Low, and lowest for High syncopation (Middle > Low > High).

#### 3.1.2 Synchrony measures (PLVs)

Analysis of PLV1 revealed significant main effects of both Syncopation and Movement condition, as well as a significant Syncopation × Movement interaction. *Post hoc* comparisons for the main effects showed higher PLV1 values for Low and Middle syncopation compared with High (Low = Middle > High), and higher PLV1 in the Dynamic condition compared with the Static and Free conditions (Dynamic > Static = Free). Inspection of the interaction patterns suggested qualitatively distinct syncopation-dependent modulation across Movement conditions: descriptively, PLV1 in the Free condition tended to peak at the Middle syncopation level, whereas in the Static condition PLV1 remained uniformly low across syncopation levels. In contrast, the Dynamic condition showed relatively high PLV1 at Low and Middle syncopation, followed by a decrease at High syncopation. For PLV2, significant main effects of Syncopation and Movement condition were observed, with no significant interaction. Post hoc comparisons indicated that PLV2 was highest for the Middle syncopation condition, with both Low and High syncopation showing lower and comparable values (Middle > Low = High). Across Movement conditions, PLV2 increased monotonically from Static to Free to Dynamic (Static < Free < Dynamic).

### 3.2 Condition-specific correlations between groove and synchrony measures

To further characterize how the relationship between groove experience and synchronization with the auditory stimulus depended on stimulus and movement context, correlation analyses were conducted separately for each combination of syncopation level and movement condition. Overall, the observed correlations were generally small in magnitude but exhibited clear condition-dependent patterns rather than uniform effects across conditions. Tables 2, 3 summarize the correlation coefficients *I*, *p*-values, and 95% confidence intervals for all combinations of movement condition and syncopation level, relating groove ratings (Urge-to-Move and Pleasure) to PLV1 (Table 2) and PLV2 (Table 3), respectively.

**Table 2:** Correlations between groove ratings and PLV1 across movement and syncopation conditions.

Movement	Syncopation	Groove	*r*	*p*	95% CI
Free	Low	Urge-to-Move	0.118	0.297	[−0.091, 0.317]
		Pleasure	0.291	< 0.01^**^	[0.089, 0.470]
	Middle	Urge-to-Move	−0.100	0.381	[−0.301, 0.109]
		Pleasure	−0.216	0.056	[−0.405, −0.009]
	High	Urge-to-Move	0.013	0.911	[−0.195, 0.219]
		Pleasure	0.070	0.535	[−0.139, 0.273]
Static	Low	Urge-to-Move	0.035	0.759	[−0.174, 0.240]
		Pleasure	0.077	0.495	[−0.132, 0.279]
	Middle	Urge-to-Move	0.001	0.995	[−0.206, 0.208]
		Pleasure	−0.022	0.848	[−0.228, 0.186]
	High	Urge-to-Move	−0.005	0.964	[−0.212, 0.202]
		Pleasure	−0.053	0.637	[−0.257, 0.156]
Dynamic	Low	Urge-to-Move	−0.087	0.441	[−0.289, 0.122]
		Pleasure	−0.028	0.807	[−0.233, 0.181]
	Middle	Urge-to-Move	−0.302	<0.01^**^	[−0.479, −0.101]
		Pleasure	−0.273	<0.05^*^	[−0.454, −0.069]
	High	Urge-to-Move	0.072	0.521	[−0.137, 0.275]
		Pleasure	0.137	0.222	[−0.072, 0.335]

Table summarizes Pearson’s correlation coefficients (*r*), *p*-values, and 95% confidence intervals (CI) between groove ratings (Urge-to-Move and Pleasure) and PLV1 for each combination of Movement condition (Free, Static, and Dynamic) and Syncopation level (Low, Middle, and High). Statistical significance is indicated by asterisks (*p < 0.05, **p < 0.01).

**Table 3:** Correlations between groove ratings and PLV2 across movement and syncopation conditions.

Movement	Syncopation	Groove	*r*	*p*	95% CI
Free	Low	Urge-to-Move	0.252	<0.05^*^	[0.047, 0.436]
		Pleasure	0.352	<0.01^**^	[0.157, 0.521]
	Middle	Urge-to-Move	0.194	0.0873	[−0.014, 0.385]
		Pleasure	0.223	<0.05^*^	[0.016, 0.411]
	High	Urge-to-Move	0.111	0.324	[−0.098, 0.311]
		Pleasure	0.007	0.953	[−0.201, 0.213]
Static	Low	Urge-to-Move	0.209	0.0616	[0.002, 0.399]
		Pleasure	0.315	<0.01^**^	[0.115, 0.490]
	Middle	Urge-to-Move	0.286	<0.01^**^	[0.084, 0.466]
		Pleasure	0.211	0.0588	[0.004, 0.400]
	High	Urge-to-Move	−0.020	0.862	[−0.226, 0.188]
		Pleasure	0.079	0.485	[−0.130, 0.281]
Dynamic	Low	Urge-to-Move	0.152	0.175	[−0.057, 0.348]
		Pleasure	0.145	0.196	[−0.064, 0.342]
	Middle	Urge-to-Move	−0.061	0.586	[−0.265, 0.147]
		Pleasure	0.082	0.465	[−0.127, 0.285]
	High	Urge-to-Move	0.112	0.319	[−0.097, 0.312]
		Pleasure	0.068	0.548	[−0.141, 0.271]

Table summarizes Pearson’s correlation coefficients (*r*), *p*-values, and 95% confidence intervals (CI) between groove ratings (Urge-to-Move and Pleasure) and PLV2 for each combination of Movement condition (Free, Static, and Dynamic) and Syncopation level (Low, Middle, and High). Statistical significance is indicated by asterisks (*p < 0.05, **p < 0.01).

For PLV1, the direction of the relationship with groove experience varied markedly across conditions. When participants listened to Low-syncopation stimuli in the Free movement condition, PLV1 showed a positive correlation with Pleasure, indicating that stronger phase locking at the slower metrical level was associated with enhanced pleasurable groove experience. In contrast, under Middle syncopation in the Dynamic movement condition, PLV1 exhibited negative correlations with both Urge-to-Move and Pleasure. A similar negative trend between PLV1 and Pleasure was also observed for the Free movement condition under Middle syncopation. These results highlight that the association between slower metrical-level synchrony and groove experience is not only condition-dependent in strength but can also reverse in direction depending on the interaction between rhythmic structure and movement context.

For PLV2, correlations with groove measures showed a broader and more consistent pattern across conditions compared to PLV1. In both Free and Static movement conditions, PLV2 tended to correlate positively with Urge-to-Move and Pleasure under Low and Middle syncopation levels, although some effects reached only trend-level significance. This pattern suggests that synchronization at the faster rhythmic timescale is more robustly associated with groove experience across a wider range of contexts, particularly when bodily movement is unconstrained or absent.

### 3.3 Embodied groove–synchrony model (SEM)

Structural equation modeling (SEM) was used to integrate the effects of musical structure, bodily movement, and auditory–motor synchrony into a unified framework, referred to as the Embodied Groove–Synchrony Model. The model was grounded in the theoretical framework proposed by Senn et al. (2019) and was designed to test how syncopation magnitude influences groove experience (Urge-to-Move and Pleasure) both directly and indirectly via movement-related synchronization processes (PLV1 and PLV2). Syncopation level (Low, Middle, and High) was incorporated simultaneously within a single model using dummy variables, as it represents graded qualitative differences in rhythmic structure within the auditory stimulus. In contrast, Movement condition (Free, Static, and Dynamic) was treated as a contextual factor that fundamentally alters the intentionality and functional role of bodily movement. Accordingly, SEM analyses were conducted separately for each Movement condition, followed by multi-group SEM to statistically compare path coefficients across conditions.

#### 3.3.1 Condition-specific SEM results

**Figure 2:**
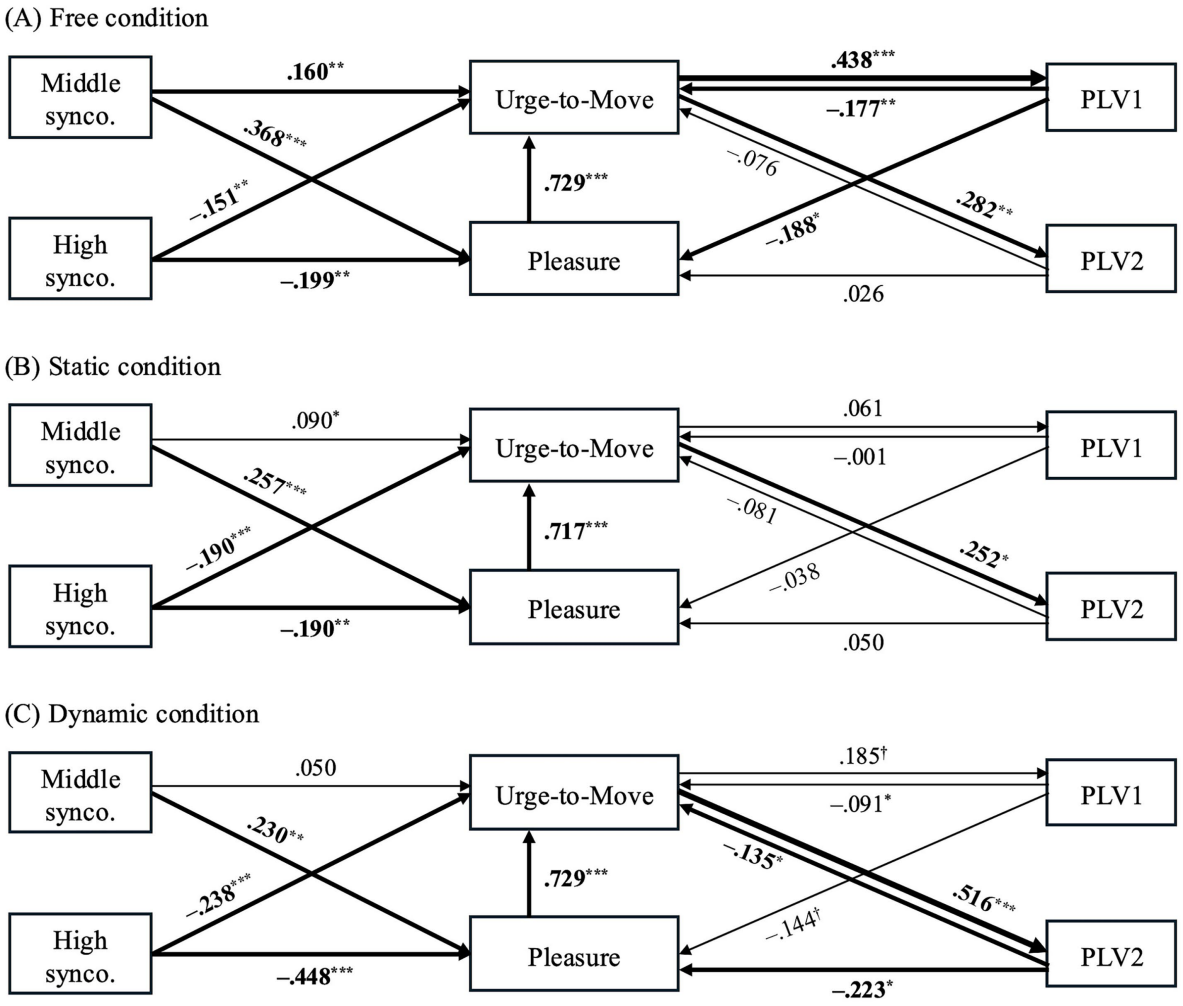
Results of the embodied groove–synchrony model (structural equation modeling). Structural equation models illustrating the relationships among musical properties (syncopation level), groove experience (urge-to-move and pleasure), and movement–music synchronization indices (PLV1 and PLV2) are shown separately for each movement condition: **(A)** Free, **(B)** Static, and **(C)** Dynamic. Syncopation level was modeled using dummy variables, with Middle synco. and High synco. Representing contrasts relative to the Low syncopation reference condition. Values on paths represent standardized path coefficients. Statistical significance of individual paths is indicated as *p < 0.05, **p < 0.01, ***p < 0.001, and trend-level effects as †*p* < 0.10. To facilitate visual interpretation, paths with absolute standardized coefficients ≥ 0.10 and *p* < 0.05 are depicted with increased line thickness, while paths with absolute coefficients ≥ 0.40 and *p* < 0.001 are shown with further increased thickness.

All three Movement conditions exhibited acceptable to good model fit (Free: CFI = 1.00, TLI = 1.02, RMSEA = 0.00, SRMR = 0.012; Static: CFI = 0.99, TLI = 0.94, RMSEA = 0.074, SRMR = 0.032; Dynamic: CFI = 0.99, TLI = 0.98, RMSEA = 0.046, SRMR = 0.022). Standardized path coefficients for all conditions are shown in Figure 2, with statistically significant paths highlighted.

##### 3.3.1.1 Free movement condition

In the Free condition, syncopation magnitude exerted differentiated effects on groove experience. Middle-level syncopation positively influenced both Urge-to-Move and Pleasure, whereas High-level syncopation exerted negative effects on both dimensions. Pleasure, in turn, further increased Urge-to-Move, indicating a reinforcing relationship between affective and motivational components of groove. Regarding embodied synchronization, Urge-to-Move positively predicted both PLV1 and PLV2, with a particularly strong association with PLV1. In contrast, PLV1 showed negative paths to both Urge-to-Move and Pleasure, suggesting that stronger phase locking at the slower metrical level constrained subjective groove experience.

##### 3.3.1.2 Static condition

In the Static condition, the effect of Middle-level syncopation on Urge-to-Move was no longer observed, while its positive effect on Pleasure remained. High-level syncopation continued to reduce both Urge-to-Move and Pleasure and Pleasure positively predicted Urge-to-Move, mirroring the Free condition. However, the coupling between groove experience and auditory–motor synchronization was markedly reduced: only a positive path from Urge-to-Move to PLV2 was observed, with no significant involvement of PLV1. This pattern indicates a restricted form of auditory–motor coupling when overt movement was suppressed.

##### 3.3.1.3 Dynamic condition

The Dynamic condition showed a pattern similar to the Static condition with respect to syncopation effects on groove experience: Middle-level syncopation selectively increased Pleasure, whereas High-level syncopation decreased both Urge-to-Move and Pleasure. Pleasure again positively predicted Urge-to-Move. In contrast to the Free condition, however, groove-related modulation of synchrony was more pronounced and shifted toward faster timescales. Urge-to-Move primarily increased PLV2, and PLV2, in turn, negatively predicted both Urge-to-Move and Pleasure, indicating a bidirectional and constraining relationship between fast-scale synchronization and groove experience.

#### 3.3.2 Multi-group SEM comparisons

Multi-group SEM comparisons revealed several condition-dependent differences in path coefficients (see Supplementary Table S1 for full statistical details). Specifically, the path from Urge-to-Move to PLV1 was significantly stronger in the Free condition than in both the Static and Dynamic conditions (Static = Dynamic < Free). The effect of High-level syncopation on Pleasure differed between Static and Dynamic conditions, with a stronger negative impact in the Dynamic condition (Dynamic < Static). Trend-level differences were observed for the path from Urge-to-Move to PLV2 between Static and Dynamic conditions (Static < Dynamic, trend), as well as for the path from PLV2 to Pleasure between Free and Dynamic conditions (Dynamic < Free, trend).

Together, these results demonstrate that while the overall structural organization of groove-related processes was preserved across movement contexts, the strength and functional role of specific synchrony pathways were systematically reshaped by movement intentionality and constraint.

#### 3.3.3 Within-subject effects of groove-synchrony pathways (LME)

To examine whether the structural relationships identified in the SEM were also present at the within-subject, trial-by-trial level, supplementary linear mixed-effects analyses were conducted. These analyses focused on pathways linking groove ratings (Urge-to-Move, Pleasure) and synchronization indices (PLV1, PLV2), while accounting for repeated observations within participants. Detailed fixed-effect estimates, degrees of freedom, and significance levels for all mixed-effects models are reported in Supplementary Table S2.

##### 3.3.3.1 Urge-to-move → PLV1/PLV2

Regarding the Urge-to-Move → PLV1 pathway, although the SEM indicated a positive association at the structural level in the Free movement condition, the LME analyses revealed no stable within-subject effect. This suggests that momentary, trial-by-trial-level fluctuations in Urge-to-Move were not reliably accompanied by corresponding changes in slower metrical-level synchrony within individuals. In contrast, the Urge-to-Move → PLV2 pathway showed a significant within-subject effect. Trials characterized by higher Urge-to-Move ratings were associated with stronger 2-Hz movement–music synchrony, indicating that this coupling operates at the level of momentary fluctuations within individuals. Moreover, this effect was modulated by Movement condition, being particularly pronounced in the Dynamic condition. This finding indicates that faster-timescale synchronization is directly sensitive to trial-by-trial variations in urge to move, particularly when movement is externally guided.

##### 3.3.3.2 PLVs → groove ratings

For the reverse pathways (PLV1/PLV2 → Urge-to-Move), the LME analyses revealed no corresponding within-subject effects, despite negative paths observed in the SEM. This dissociation indicates that the inhibitory influence of synchronization on groove experience reflects a higher-level regulatory structure across conditions rather than immediate trial-by-trial modulation. A similar dissociation was observed for PLV → Pleasure. While the SEM showed a negative PLV1 → Pleasure path in the Free condition, the LME analyses instead revealed a positive within-subject coupling specifically in Free condition. This suggests that, although excessive synchronization may constrain pleasure at the structural level, transient increases in slower metrical-level synchrony can locally enhance pleasurable experience during free movement. In contrast, PLV2 showed a significant positive within-subject effect on Pleasure across conditions, indicating a more consistent facilitatory role of faster timescale synchronization at the trial level.

Taken together, these results indicate that among the groove–synchrony pathways identified in the SEM, only the Urge-to-Move → PLV2 relationship is consistently expressed at the level of within-subject, trial-by-trial variability. Other pathways appear to characterize stable, condition-dependent organizational principles of embodied groove rather than immediate dynamic coupling.

## 4 Discussion

### 4.1 Overview of the main findings

The present study investigated how musical structure, bodily movement, and auditory–motor synchronization jointly shape the experience of groove, with a particular focus on the movement-related context in which these relationships unfold. By combining ANOVAs, condition-specific correlation analyses, structural equation modeling (SEM), and supplementary linear mixed-effects models (LME), we examined not only whether groove-related variables are associated, but how their functional organization changes depending on whether and how listeners are allowed to move.

At the behavioral level, ANOVA results replicated a well-established finding in groove research: both Urge-to-Move and Pleasure were maximized at moderate levels of syncopation, consistent with previous reports of an inverted U-shaped relationship between syncopation and groove (e.g., Witek et al., 2014; Stupacher et al., 2022b). Movement condition further modulated these effects; Urge-to-Move was significantly enhanced in the Dynamic condition relative to the Static condition. Notably, Pleasure did not show a main effect of movement condition, despite prior findings that moving to music can enhance affective experience (e.g., Bernardi et al., 2017). This suggests that pleasurable aspects of groove in the present paradigm were primarily driven by musical structure—particularly syncopation—rather than by the mere permissibility or presence of bodily movement. This pattern further highlights a functional dissociation between pleasure and urge-to-move: although tightly coupled, only the latter was systematically shaped by the movement context, pointing to potentially distinct roles of affective valuation and action-oriented motivation within the groove experience. Synchronization measures likewise showed differentiated sensitivities to musical and movement-related factors. While both PLV1 and PLV2 were modulated by syncopation and movement condition, they exhibited distinct patterns of association with groove-related ratings. Specifically, PLV2 tended to show relatively consistent positive correlations with Urge-to-Move across conditions, whereas PLV1—reflecting slower, more global synchronization—displayed more variable relationships, including condition- and syncopation-dependent reversals in correlation sign. Condition-specific correlation analyses thus indicated that auditory–motor synchronization does not contribute uniformly to groove experience, but relates to Urge-to-Move and Pleasure in qualitatively different ways depending on temporal scale and movement context. Although movement amplitude differed across movement conditions, reflecting task instructions, it did not vary systematically with syncopation, suggesting that groove-related effects are not simply driven by the magnitude of movement. Taken together, these findings suggest that the relationship between synchronization and groove varies across levels of syncopation. Therefore, collapsing across syncopation levels may obscure condition-specific patterns, particularly given the non-linear nature of groove responses to rhythmic complexity.

These descriptive findings were integrated and extended by the SEM analyses, which provided a formal account of how musical structure, groove experience, and synchronization are functionally organized within each movement condition. Across all conditions, the Embodied Groove–Synchrony Model showed acceptable to good fit, supporting its validity as a unifying framework. Crucially, the SEM revealed a consistent asymmetry in directional effects: groove experience—especially Urge-to-Move—positively predicted synchronization, whereas synchronization exerted negative effects on Urge-to-Move and/or Pleasure. This pattern contrasts with psychological models that conceptualize entrained body movement primarily as a facilitator of groove experience, in which bodily synchronization enhances pleasure, energetic arousal, and movement motivation (e.g., Senn et al., 2019, 2023). Instead, the present results suggest that synchronization may also play a constraining or regulatory role within the groove experience. Importantly, the specific pathways and their strengths differed across movement conditions, indicating that the functional organization of groove is reshaped by the intentionality and permissibility of bodily movement.

Supplementary LME analyses further clarified which of these relationships also operate at the within-subject, trial-by-trial level. Among the tested pathways, only the effect of Urge-to-Move on PLV2 showed a reliable within-subject association, indicating that momentary increases in groove-related impulse are accompanied by stronger 2-Hz synchronization, particularly in the Dynamic condition. In contrast, the negative effects of synchronization on groove observed in the SEM were not reflected in trial-level fluctuations. This dissociation suggests that these negative pathways capture condition-level regulatory structures rather than immediate causal effects. That is, they likely reflect relatively stable, between-subject differences within each movement condition—such as individuals’ preferred synchronization strategies, habitual attentional focus to the musical structure, or characteristic movement stabilization—rather than trial-by-trial fluctuations within the same individual. Taken together, these results indicate that groove is not simply amplified by bodily synchronization, but is dynamically organized through context-dependent feedback between musical structure, movement intention, and synchronization.

### 4.2 Movement context and synchronization jointly shape the groove experience

The present results demonstrate that groove experience emerges from an interaction between musical structure, bodily movement, and auditory–motor synchronization, rather than from any single factor in isolation. Crucially, the movement context in which listeners engage with music systematically reshaped how synchronization relates to subjective groove components. These findings indicate that the functional role of synchronization within groove experience is context-dependent, varying according to whether bodily movement is permitted, constrained, or absent. It should also be noted that the present PLV measure reflects synchronization to a reference temporal structure (i.e., canonical frequency components such as 1 or 2 Hz), rather than direct phase coupling to the acoustic signal itself. This suggests that the observed synchronization may partly reflect alignment to internally represented temporal structure (e.g., perceived beat or metrical hierarchy), rather than purely stimulus-driven tracking of acoustic features.

These findings can also be interpreted within broader theoretical frameworks of predictive processing, entrainment, and embodied music cognition. From a predictive processing perspective, bodily movement may reflect active inference, whereby listeners continuously generate and update temporal predictions about incoming rhythmic structure (Clark, 2015). Within this framework, synchronization reflects a balance between prediction and error correction: stronger phase-locking may indicate stabilized predictions, whereas reduced synchronization may reflect adaptive flexibility and ongoing prediction updating. This interpretation is also consistent with entrainment theory, which characterizes synchronization as the dynamic alignment of internal and external rhythms (Clayton, 2012), and with embodied music cognition accounts emphasizing action–perception coupling (Leman, 2008). Furthermore, dynamic attending theory suggests that attentional resources are rhythmically modulated and can shift across hierarchical temporal levels (Large and Jones, 1999), providing a potential account for the observed differences between 1 and 2 Hz synchronization. While the present study was not designed to directly test these frameworks, the results are broadly consistent with the view that groove emerges from a dynamic interplay between prediction, movement, and temporally structured attention.

#### 4.2.1 Movement context as a modulator of groove–synchrony coupling

The relationship between syncopation, groove experience, and auditory–motor synchronization was strongly contingent on movement context, revealing that neither musical structure nor synchronization exerts uniform effects across conditions. In the Free and Static conditions, PLV2 showed positive correlations with groove ratings under Low and Middle syncopation levels, whereas no such correlations were observed in the Dynamic condition (Table 3). This dissociation suggests that the expression of groove–synchrony coupling depends critically on whether movement is spontaneous, suppressed, or intentionally executed. In the Dynamic condition, the absence of clear correlations can be explained by the presence of opposing directional pathways identified in the SEM. Specifically, Urge-to-Move exerted a positive influence on PLV2, while PLV2 simultaneously exerted a negative influence on Urge-to-Move (Figure 2). The coexistence of these counteracting effects likely attenuated their net expression at the correlational level, obscuring simple associations despite a structured bidirectional organization. In contrast, in the Free and Static conditions—where such antagonistic pathways were weaker or absent—PLV2 more directly indexed groove-related engagement, particularly when rhythmic complexity remained within a moderate range.

In addition, movement context shaped how syncopation influenced groove experience itself. A direct positive effect of Middle syncopation on Urge-to-Move was observed exclusively in the Free condition (Figure 2). This indicates that moderate prediction error translated directly into movement motivation only when listeners were free to regulate their bodily engagement. In the Static and Dynamic conditions, Middle syncopation did not directly increase Urge-to-Move; instead, its influence was mediated via Pleasure. This pattern suggests that when movement is constrained or prescribed, rhythmic complexity primarily modulates affective evaluation rather than directly eliciting motor impulse. At higher levels of rhythmic complexity, further context-dependent divergences emerged. High syncopation exerted a significantly stronger negative effect on Pleasure in the Dynamic condition compared to the other conditions. While intentional movement to music—such as dancing—has been shown to enhance affective experience under appropriate circumstances (e.g., Bernardi et al., 2017), the present results suggest that such benefits depend on the predictability of the musical structure. When listeners are required to move in synchrony with highly unpredictable rhythms, the cognitive and sensorimotor demands may outweigh the hedonic benefits of movement, leading to a disproportionate reduction in Pleasure. This finding highlights a boundary condition for the commonly assumed positive relationship between movement and musical enjoyment.

Across all three conditions, Urge-to-Move consistently increased PLV2, and in the Static condition this was the only significant synchronization-related pathway. This convergence suggests that the impulse to move itself robustly induces synchronization at approximately 2 Hz, a timescale that falls within the range of natural rhythmic behaviors such as stepping or head movements (approximately 1–3 Hz), and that aligns with delta-band neural dynamics implicated in auditory–motor coupling rather than reflecting a fixed movement frequency (Doelling et al., 2023). Prior work has linked groove-related movement tendencies to the coupling between auditory rhythms and motor timing networks, including basal ganglia–cortical circuits and motor predictive mechanisms (e.g., Vuust and Witek, 2014; Janata et al., 2012), providing a plausible neural substrate for this effect. However, the functional role of synchronization differed markedly across movement contexts. In the Static condition, synchronization did not feed back onto groove experience. Because bodily motion was minimized, overall synchronization levels were low (Table 1), effectively abolishing any meaningful interaction between synchronization and subjective groove. In contrast, the Free and Dynamic conditions showed selective interactions with different temporal scales of synchronization: the Free condition was primarily associated with 1-Hz synchronization, whereas the Dynamic condition interacted more strongly with 2-Hz synchronization (Figure 2). This dissociation may reflect differences in movement strategy and attentional focus. That is, Free movement likely permits slower, whole-body entrainment aligned with higher-level metrical structure, whereas the Dynamic condition encourages faster, repetitive movements tightly coupled to the beat cycle, thereby emphasizing synchronization at shorter temporal scales.

Together, these results demonstrate that movement context fundamentally reorganizes the functional relationships among syncopation, groove experience, and synchronization. Rather than acting as a uniform enhancer of groove, synchronization emerges as a context-sensitive process whose temporal scale, directionality, and experiential consequences depend on how listeners are allowed—or required—to move.

#### 4.2.2 Synchronization is not always beneficial: when entrainment constrains groove

A central contribution of the present study lies in demonstrating that auditory–motor synchronization does not uniformly enhance the groove experience. This finding calls for a careful reconsideration of how “entrained body movement” has been conceptualized in existing psychological models of groove, most notably that of Senn et al. (2023). In their framework, entrained body movement is treated as a qualitative, experiential, and action-oriented construct, encompassing overt movement, felt engagement, and the subjective sense of being “carried” by the music. By contrast, the present study operationalized synchronization using phase-locking value (PLV), a quantitative and timing-specific index that captures the degree of temporal stabilization between bodily signals and musical structure. These two notions thus refer to different analytical levels of movement: entrained body movement in the sense of Senn et al. (2023) reflects a phenomenological and behavioral level of embodied engagement, whereas auditory–motor synchronization as quantified by PLV captures a dynamical and coordinative level of temporal coupling between movement and sound. This distinction is crucial for interpreting the observed negative effects of synchronization on groove-related experience. Across the Free and Dynamic movement conditions, the SEM consistently revealed negative paths from PLV to Urge-to-Move and/or Pleasure, indicating that stronger phase alignment was associated with a reduction in subjective groove. This pattern represents a decisive departure from the Senn model, in which entrained body movement is assumed to feedback positively into pleasure, energetic arousal, and the urge to move. Rather than functioning solely as a facilitator, synchronization in the present paradigm appears to play a constraining or regulatory role, suggesting that groove involves not only coupling per se, but also the preservation of sensorimotor flexibility that allows ongoing prediction and adjustment. In this context, it is also important to consider that, particularly in syncopated rhythms, the temporal alignment between acoustic events and the perceived beat may be partially dissociated. As a result, synchronization to a canonical temporal reference (as measured by PLV in the present study) may not fully capture alignment with surface rhythmic events. This dissociation may contribute to the variability, and, in some cases, negative associations observed between synchronization and groove-related responses. To further examine this interpretation, we conducted a supplementary participant-level analysis relating mean synchronization strength to mean groove-related ratings within each condition. No significant correlations were observed between synchronization and either Urge-to-Move or Pleasure (all |*r*| < 0.10, all *p* > 0.65). This absence of simple bivariate associations suggests that the effects identified in the SEM do not reflect direct pairwise relationships, but instead emerge from the multivariate structure of the model, in which synchronization interacts with other factors within the broader system to shape groove experience.

Importantly, these findings do not imply that bodily movement per se diminishes groove. Instead, the results point to a more specific claim: entrained body movement is not equivalent to high phase-locking. Whereas movement can be expressive, exploratory, and variable, high PLV reflects a state of temporal over-organization or stabilization. From this perspective, excessively ordered synchronization may dampen the very tension, ambiguity, or play between prediction and violation that is thought to underlie the groove experience (e.g., Vuust et al., 2022). In other words, synchronization that becomes too rigid may attenuate groove by reducing the space for embodied prediction, adjustment, and expressive micro-variation. Indeed, phase synchronization of neural oscillations has been proposed to reflect functional stabilization and top–down control of information processing (Engel et al., 2001; Fries, 2015). From a dynamical systems perspective, excessive stabilization or overly strong synchronization can reduce behavioral and perceptual flexibility, thereby constraining adaptive coordination (Kelso, 1995; Delignières and Marmelat, 2014). Consistent with these views, previous work on rhythm perception and musical groove suggests that a certain degree of temporal variability or fluctuation—rather than perfectly regular timing—plays a crucial role in sustaining engagement and embodied musical experience (Large and Palmer, 2002; Hennig, 2014). This interpretation aligns with the broader idea that groove thrives not at extremes of order or disorder, but within a dynamically balanced regime.

The supplementary linear mixed-effects analyses further support this interpretation by clarifying the temporal scale at which these relationships operate. Among all tested pathways, only the positive effect of Urge-to-Move on PLV2 was reliably observed at the within-subject, trial-by-trial level across all movement conditions. This suggests that momentary increases in the impulse to move directly promote stronger synchronization at the 2-Hz timescale. In contrast, the negative effects of PLV on Urge-to-Move and Pleasure did not manifest as within-subject fluctuations, indicating that they are better understood as reflecting between-subject or condition-level tendencies. That is, individuals who, on average, exhibit stronger synchronization within a given movement context tend to report lower levels of groove-related experience. Such effects likely capture relatively stable synchronization strategies, attentional orientations toward timing accuracy, or movement stabilization tendencies, rather than immediate causal suppression of groove.

Finally, these findings bear directly on the unresolved question of the causal relationship between Urge-to-Move and Pleasure highlighted in Etani et al. (2024). Although these two components of groove are strongly correlated, their directional relationship remains unclear. In the present study, attempts to extend the SEM by adding a path from Urge-to-Move to Pleasure resulted in model non-convergence across all movement conditions. This instability suggests that the relationship between urge to move and pleasure cannot be straightforwardly reduced to a unidirectional causal chain, and that additional mediating or moderating processes—such as synchronization dynamics or movement context—must be taken into account. Together, these results reinforce the view that groove is not simply amplified by tighter synchronization, but is shaped by a delicate interplay between movement impulse, temporal coordination, and the contextual constraints under which bodily engagement with music unfolds.

#### 4.2.3 Interpreting context-dependent groove through embodied and predictive frameworks

Music perception is tightly coupled with the motor system. Even in the absence of overt movement, merely listening to rhythmic music reliably activates motor-related regions such as the supplementary motor area (SMA), premotor cortex, and basal ganglia (Grahn and Brett, 2007; Chen et al., 2008; Bengtsson et al., 2009). Furthermore, listening to high-groove music has been shown to engage reward-related regions including the nucleus accumbens, caudate nucleus, and medial orbitofrontal cortex (mOFC), alongside areas involved in beat perception and motor timing such as the putamen and SMA, as well as prefrontal and parietal cortices (Matthews et al., 2020). These findings suggest that groove experience emerges from coordinated activity across auditory, motor, attentional, and reward-related systems, even when no explicit movement is required.

Within this broader neurocognitive context, the present finding that Urge-to-Move consistently increased PLV2 across all movement conditions (Figure 2) suggests that groove-related motor motivation actively shapes auditory–motor synchronization. This common pathway indicates that the urge to move is sufficient to enhance synchronization at the movement-related temporal scale (i.e., 2 Hz), independent of whether overt movement is permitted or constrained. Evidence that high-groove music enhances delta-band neural entrainment and attentional engagement (Cameron et al., 2019) further supports the interpretation that increased PLV reflects an attentive, action-oriented state in which auditory and motor systems are more tightly coordinated. In this sense, increases in PLV do not merely index incidental movement-related synchronization, but rather reflect a motivated and attentive state in which auditory–motor networks are more tightly coordinated.

Importantly, predictive processing frameworks offer a principled account of why the experiential consequences of synchronization differ across movement contexts. Predictive coding theories propose that perception and action jointly serve to minimize prediction error, either by updating internal models or by generating movements that stabilize sensory input (Friston, 2010; Vuust et al., 2022). In the Dynamic condition, intentional and repetitive movement likely imposes strong constraints on temporal prediction at the movement-related timescale. Under such conditions, increased PLV2 may reflect a stabilization strategy aimed at reducing temporal uncertainty. While this strategy supports precise coordination, it may simultaneously constrain groove experience by limiting flexibility in prediction, adjustment, and expressive micro-variation. By contrast, in the Free condition, groove experience was more strongly linked to synchronization at the slower metrical timescale (PLV1). This suggests that when movement is self-directed and exploratory, the motor system supports groove primarily by flexibly aligning with the musical pulse rather than by enforcing rigid timing at a specific movement frequency. In this context, the motor system may facilitate groove by enabling flexible alignment with the musical pulse, rather than by enforcing rigid temporal regularity. The dissociation between PLV1-dominated control in the Free condition and PLV2-dominated control in the Dynamic condition thus indicates that increased motor engagement does not uniformly enhance groove, but instead reshapes the temporal level at which groove is regulated.

Taken together, these findings highlight that groove cannot be reduced to the strength of auditory–motor synchronization alone. Rather, groove emerges from a context-dependent balance between synchronization and flexibility within embodied predictive processes. While high-groove music robustly engages motor and reward systems and enhances neural entrainment and attention, excessive stabilization—particularly under conditions of intentional movement—may constrain rather than amplify groove experience. Synchronization, therefore, functions not simply as a facilitator of groove, but as a regulatory mechanism whose experiential impact depends on movement context and temporal scale.

### 4.3 Limitations and future directions

Several limitations of the present study should be acknowledged, alongside promising avenues for future research. First, the participant sample consisted of Japanese listeners with a relatively homogeneous level of amateur musical experience. Previous studies have demonstrated that musical training can qualitatively shape groove experience, including sensitivity to syncopation and the coupling between movement and pleasure (e.g., Matthews et al., 2019, 2022; Senn et al., 2018). Although the present sampling strategy was adopted to ensure a consistent inverted U-shaped relationship between syncopation and groove, future studies should examine whether the observed movement–synchrony–groove organization generalizes across different levels of musical expertise. Moreover, cultural differences in music-related bodily expression and rhythmic entrainment should be considered when interpreting the present findings. Entrainment to musical rhythm is not a purely universal phenomenon but is shaped by culturally learned listening practices and movement conventions (Clayton et al., 2005). In addition, culturally situated traditions—such as those emphasizing expressive microtiming and embodied rhythmic participation—highlight that movement-related engagement with music can be learned and internalized in culturally specific ways (Iyer, 2002). Given these considerations, the present results, obtained from Japanese participants, may reflect culturally contingent modes of groove experience and auditory–motor coordination, underscoring the need for future cross-cultural investigations.

Second, movement assessment in the present study was limited to head motion, and in the Dynamic condition participants were explicitly instructed to move their head. Head movement is a robust and commonly observed manifestation of groove (Janata et al., 2012; Dotov et al., 2021), yet spontaneous groove-related movement can also involve other body parts, such as foot tapping or finger movements, particularly in less constrained contexts. Future work should therefore incorporate whole-body motion capture and examine synchronization across multiple body segments, enabling a more comprehensive characterization of embodied groove.

Beyond timing accuracy and phase coupling, groove experience is closely intertwined with expressive bodily movement, particularly dance-like whole-body engagement. Previous studies have shown that high-groove music reliably induces spontaneous movement across multiple body segments, and that such movement is not merely a byproduct of listening but contributes to how music is experienced (Janata et al., 2012; Dotov et al., 2021). Importantly, dance-related movement provides a richer form of embodied engagement than isolated actions such as tapping, involving coordination across the trunk, limbs, and balance control systems. Research on music-induced dance has demonstrated that groove-related movement patterns reflect both rhythmic structure and expressive interpretation, rather than strict temporal alignment alone (Toiviainen et al., 2010; Burger et al., 2013). These findings suggest that groove may emerge most strongly when listeners are able to translate rhythmic affordances into flexible, expressive bodily dynamics, rather than when movement is constrained to a single body part or reduced to timing precision. Accordingly, future studies should investigate how dance-like whole-body expression modulates the relationship between synchronization, pleasure, and urge to move, and whether different movement repertoires give rise to qualitatively distinct groove experiences.

Finally, an important direction for future research concerns the distinction between objectively measured synchronization and subjectively perceived synchrony. Prior work has shown that perceived synchrony is more strongly associated with groove ratings than objective tapping accuracy, particularly for rhythms with medium syncopation (Matthews et al., 2022). This discrepancy suggests that groove experience may be more closely linked to listeners’ internal sense of alignment with the music than to precise temporal correspondence. Collecting subjective synchrony ratings alongside objective synchronization measures would therefore allow future studies to disentangle perceptual, motor, and experiential contributions to groove, and to clarify their causal relationships.

### 4.4 Conclusion

This study examined how musical structure, bodily movement, and auditory–motor synchronization jointly shapes the experience of groove, with a particular focus on movement context. By combining behavioral ratings with multiscale synchronization measures and condition-specific modeling, we showed that groove is not a simple outcome of entrained movement, but a context-dependent phenomenon emerging from interactions between urge to move, pleasure, and synchronization.

Across conditions, Urge-to-Move consistently predicted stronger synchronization at the 2-Hz timescale, highlighting its central role in driving auditory–motor coupling. At the same time, synchronization did not uniformly enhance groove. In Free and Dynamic conditions, stronger phase-locking was associated with reduced Urge-to-Move and/or Pleasure, suggesting that overly stabilized synchronization can constrain groove rather than facilitate it. These findings challenge models that conceptualize entrained body movement as an exclusively positive contributor to groove.

Importantly, the functional organization of groove varied with movement context. Moderate syncopation directly increased Urge-to-Move only when movement was unconstrained, and different temporal scales of synchronization were implicated across conditions. Together, these results support the Embodied Groove–Synchrony Model, which emphasizes that groove arises from a balance between prediction, movement, and flexibility, rather than from maximal entrainment alone. Understanding groove therefore requires considering not only whether listeners synchronize to music, but how and under what movement constraints this synchronization occurs.

## Data Availability

The raw data supporting the conclusions of this article will be made available by the authors, without undue reservation.
