# Supplementary material for: Embodied groove–synchrony model: movement context reshapes groove–synchrony coupling and its dominant timescale
Source: Front Psychol. 2026 May 15;17:1803480. doi: 10.3389/fpsyg.2026.1803480 (PMC13219280; doi:10.3389/fpsyg.2026.1803480)
Supplement: Supplementary file 1 [file Supplementary_file_1.DOCX]

Supplementary Material

# Supplementary Tables

**Supplementary Table S1.** Multi-group SEM comparisons of path coefficients across Movement conditions (Free, Static, Dynamic). Results of multi-group structural equation modeling (SEM) comparing standardized path coefficients across Movement conditions (Free, Static, Dynamic). For each path, chi-squared difference tests (Δχ²) were computed by selectively releasing equality constraints across groups. Significant or trend-level effects are summarized using post hoc comparisons indicating the relative ordering of path strengths between conditions; all other paths are labeled as n.s. (not significant). Synco_M and Synco_H denote dummy variables for Middle and High syncopation levels, respectively, with Low syncopation as the reference category. *p < .05, **p < .01; † p < .10.

| **Path** | **Δχ²** | **df** | **p-value** | **Post hoc comparisons** |
| --- | --- | --- | --- | --- |
| Synco_M → Urge-to-Move | 2.70 | 2 | 0.26 | n.s. |
| Synco_M → Pleasure | 2.23 | 2 | 0.33 | n.s. |
| Synco_H → Urge-to-Move | 2.46 | 2 | 0.29 | n.s. |
| Synco_H → Pleasure | 9.48 | 2 | 0.0087^**^ | Dynamic < Static |
| Pleasure → Urge-to-Move | 0.68 | 2 | 0.71 | n.s. |
| Urge-to-Move → PLV1 | 6.38 | 2 | 0.041^*^ | Static = Dynamic < Free |
| Urge-to-Move → PLV2 | 5.74 | 2 | 0.057^†^ | Static < Dynamic |
| PLV1 → Urge-to-Move | 3.19 | 2 | 0.20 | n.s. |
| PLV1 → Pleasure | 0.64 | 2 | 0.73 |  |
| PLV2 → Urge-to-Move | 0.76 | 2 | 0.69 | n.s. |
| PLV2 → Pleasure | 4.73 | 2 | 0.094^†^ | Dynamic < Free |

**Supplementary Table S2.** Summary of supplementary linear mixed-effects analyses examining within-subject (trial-by-trial) effects corresponding to SEM paths. Fixed effects from linear mixed-effects models examining within-subject (trial-by-trial) associations between groove ratings (Urge-to-Move, Pleasure) and auditory–movement synchrony measures (PLV1, PLV2). All models included random intercepts for participants. Interaction terms are reported separately by Movement condition when applicable. *p < .05, **p < .01; † p < .10.

| **(A) Urge-to-Move → PLV1** | | | | | | |
| --- | --- | --- | --- | --- | --- | --- |
| **Predictor** | **Movement condition** | **β** | **SE** | **df** | **t** | **p-value** |
| Urge-to-Move | Overall | 0.0099 | 0.0080 | 703.28 | 1.24 | 0.22 |
| Urge-to-Move × Movement (Free) | Free | 0.012 | 0.011 | 697.29 | 1.10 | 0.27 |
| Urge-to-Move × Movement (Dynamic) | Dynamic | −0.0019 | 0.011 | 698.52 | −0.18 | 0.86 |
| **(B) Urge-to-Move → PLV2** | | | | | | |
| **Predictor** | **Movement condition** | **β** | **SE** | **df** | **t** | **p-value** |
| Urge-to-Move | Overall | 0.023 | 0.0092 | 701.79 | 2.48 | 0.014^*^ |
| Urge-to-Move × Movement (Free) | Free | 0.022 | 0.013 | 696.46 | 1.72 | 0.087^†^ |
| Urge-to-Move × Movement (Dynamic) | Dynamic | 0.026 | 0.013 | 697.54 | 2.05 | 0.040^*^ |
| **(C) PLV1/PLV2 → Urge-to-Move** | | | | | | |
| **Predictor** | **Movement condition** | **β** | **SE** | **df** | **t** | **p-value** |
| PLV1 | Overall | 0.28 | 0.39 | 701.29 | 0.72 | 0.47 |
| PLV2 | Overall | 0.27 | 0.32 | 707.13 | 0.84 | 0.40 |
| **(D) PLV1/PLV2 → Pleasure** | | | | | | |
| **Predictor** | **Movement condition** | **β** | **SE** | **df** | **t** | **p-value** |
| PLV1 | Overall | −0.36 | 0.67 | 701.41 | −0.53 | 0.59 |
| PLV1 × Movement (Free) | Free | 1.99 | 0.82 | 705.76 | 2.42 | 0.016^*^ |
| PLV2 | Overall | 1.35 | 0.54 | 708.61 | 2.52 | 0.012^*^ |
